# A Systematic Comparison of Task Adaptation Techniques for Digital Histopathology

**DOI:** 10.3390/bioengineering11010019

**Published:** 2023-12-24

**Authors:** Daniel Sauter, Georg Lodde, Felix Nensa, Dirk Schadendorf, Elisabeth Livingstone, Markus Kukuk

**Affiliations:** 1 Department of Computer Science, Fachhochschule Dortmund, 44227 Dortmund, Germany; markus.kukuk@fh-dortmund.de; 2 Department of Dermatology, University Hospital Essen, 45147 Essen, Germany; georg.lodde@uk-essen.de (G.L.); dirk.schadendorf@uk-essen.de (D.S.); elisabeth.livingstone@uk-essen.de (E.L.); 3 Institute for AI in Medicine (IKIM), University Hospital Essen, 45131 Essen, Germany; felix.nensa@uk-essen.de; 4 Institute of Diagnostic and Interventional Radiology and Neuroradiology, University Hospital Essen, 45147 Essen, Germany

**Keywords:** transfer learning, fine-tuning, computer vision, CNN, whole-slide imaging, cancer

## Abstract

Due to an insufficient amount of image annotation, artificial intelligence in computational histopathology usually relies on fine-tuning pre-trained neural networks. While vanilla fine-tuning has shown to be effective, research on computer vision has recently proposed improved algorithms, promising better accuracy. While initial studies have demonstrated the benefits of these algorithms for medical AI, in particular for radiology, there is no empirical evidence for improved accuracy in histopathology. Therefore, based on the ConvNeXt architecture, our study performs a systematic comparison of nine task adaptation techniques, namely, DELTA, L^2^-SP, MARS-PGM, Bi-Tuning, BSS, MultiTune, SpotTune, Co-Tuning, and vanilla fine-tuning, on five histopathological classification tasks using eight datasets. The results are based on external testing and statistical validation and reveal a multifaceted picture: some techniques are better suited for histopathology than others, but depending on the classification task, a significant relative improvement in accuracy was observed for five advanced task adaptation techniques over the control method, i.e., vanilla fine-tuning (e.g., Co-Tuning: *P*(≫) = 0.942, *d* = 2.623). Furthermore, we studied the classification accuracy for three of the nine methods with respect to the training set size (e.g., Co-Tuning: *P*(≫) = 0.951, *γ* = 0.748). Overall, our results show that the performance of advanced task adaptation techniques in histopathology is affected by influencing factors such as the specific classification task or the size of the training dataset.

## 1. Introduction

The need for improved tools in clinical histopathology has been repeatedly emphasized. Commonly cited arguments include an increasing need for accuracy, a lack of specialized histopathologists, subjectivity, and a lack of reproducibility [1,2]. Furthermore, the situation is expected to worsen due to increasing cancer incidence and more screening campaigns [3]. Fortunately, deep neural networks are expected to significantly change the study of cancer tissue in histopathology [1]. In fact, deep learning (DL) in computational histopathology promises various benefits. These include time and cost savings, lower error rates, better accessibility, and the learning of more accurate representations [2]. Furthermore, it can improve clinical decision making by providing access to high-level information [4]. It also plays an important role in mining big data [5]. As a result, the yearly number of research papers on DL-based histopathology is steadily increasing [3,6].

However, DL models must achieve a sufficient level of accuracy to be approved for clinical practice, while digital histopathology poses several challenges for the application of DL, including large image size, insufficient data annotation, varying magnification levels of the microscope, staining artifacts, and color variation [2]. Transfer learning, including the fine-tuning of pre-trained models, is a standard technical solution for insufficient annotation. Given limited training data, fine-tuning can significantly improve the performance compared to training from scratch [7]. Kornblith et al. [8] also found that, on average, fine-tuning achieved a 17-fold increase in the training speed compared to training from scratch. This was confirmed by He et al. [9]. In histopathology, fine-tuning appears to be generally superior to using off-the-shelf features [10,11].

The exact procedure for fine-tuning, however, is still an active field of research. Early studies attempted to systematically evaluate the relationship between model parameters and transferability in convolutional neural networks (CNNs) [12]. Recently, several studies have tried to systematically investigate the impact of pre-training [13,14], algorithms [15,16], and parameters [8,17]. Raghu et al. [18] reported findings on model size in the medical domain. A recent literature review for computer vision (CV) with further information can be found in the dissertation by Plested [19], among others.

Especially the technical approach to transfer learning in histopathology leaves room for improvement. The most straightforward algorithm consists of copying the pre-trained weights of the initial layers, reinitializing the subsequent layers, and then training them together. This algorithm dates back to the early 2010s [7,20,21]. At present, researchers using pre-trained DL models for digital histopathology can choose from various recently proposed algorithms from CV research [22,23,24]. Some initially successful applications of advanced transfer learning techniques in the medical domain can be found with respect to radiological images [25,26,27,28] or lung sound analysis [29]. However, the wider adoption of these techniques in medicine has not occurred to date. For example, “task adaptation,” which transfers a pre-trained model into a supervised target domain [30], has not been widely explored for histopathology. In general, Sauter et al. [31] recently noticed a lack of transfer of state-of-the-art DL methodology into the histopathology of malignant melanoma.

On the other hand, choosing a transfer learning algorithm for histopathology can be challenging. It is known that no machine learning (ML) algorithm is generally superior in every conceivable use case [32]. More specifically, Zhang et al. [33] noted that, in addition to other factors, the benefit of transfer learning depends on the algorithm due to assumptions made and specific application scenarios. Accordingly, the question arises of whether transfer learning algorithms that perform well on datasets like ImageNet are also suitable for histopathology. In addition, there is the related question of how much each algorithm benefits from more labeled training data. From a practical point of view, gaining only a small increase in accuracy would perhaps speak against the labor- and cost-intensive effort of labeling additional training data by pathology experts [2]. Systematic evaluations of other technical aspects, such as pre-training [34,35] and data augmentation [36], exist. However, there is—to our knowledge—no systematic comparison of task adaptation strategies applied to histopathological data.

The present study intends to advance the development of DL models for histopathology via improved transfer learning techniques from CV. Based on our findings, follow-up research can enhance their models addressing specific clinical questions and achieve accuracy levels sufficient for clinical approval. This will hopefully help to realize the potential benefits of DL in clinical patient care. Therefore, in our study, we would like to know which task adaptation technique(s) should be used when developing DL models for medical use cases to achieve the best possible accuracy. Consequently, our research question is as follows: which task adaptation technique achieves the highest accuracy (in terms of AUC) for classification in digital histopathology tasks?

Our study has several unique contributions, which can be summarized as follows:

- It is the first systematic evaluation of up-to-date task adaptation techniques for classification tasks in digital histopathology.
- We comprehensively carried out evaluations based on five histopathological classification tasks (mitotic figure detection, tumor metastasis detection, tumor-infiltrating lymphocytes detection, colorectal cancer tissue-type classification, and skin cancer tissue-type classification) and eight datasets. Our evaluations include external testing and statistical validation.
- We show that the standard fine-tuning procedure can be outperformed by more advanced task adaptation techniques depending on the task at hand.
- Furthermore, the impact of dataset size is investigated. We show that the Co-Tuning technique can offer further improvements in large-scale settings.

## 2. Research Background

The following section that presents the research background is divided into two parts. First, the section introduces some categorization schemas of transfer learning. Second, techniques from the literature on CV are described and categorized from a technical perspective.

### 2.1. Transfer Learning

**Figure 1:**
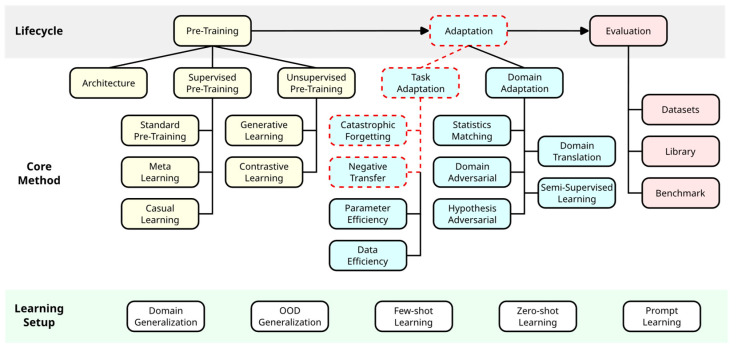
Lifecycle-oriented perspective on transfer learning. Transfer learning includes pre-training on an upstream task in the source domain and adaptation to a downstream task in the target domain. The adaptation process can be divided based on whether labels are available in the target domain (“task adaptation”) or not (“domain adaptation”). Red dashed lines highlight the subcategories examined in our study. Adapted with permission from Jiang et al. [30].

Transfer learning is a technique in the field of ML that has been studied for some time. Its goal is to improve ML models by transferring knowledge from a source domain to the target domain [37]. Jiang et al. [30] recently structured research on transfer learning for DL from a lifecycle-oriented perspective (see Figure 1). The main training steps are pre-training a model in the source domain to learn transferable knowledge and then adapting it in the target domain. In the adaptation step, a further distinction is made between whether labels are available in the target domain (“task adaptation”) or not (“domain adaptation”) [30]. Task adaptation is further divided into four subcategories. “Catastrophic forgetting” refers to the loss of information that was learned from previous tasks when subsequently learning a new task [38,39]. “Negative transfer” describes a decrease in performance in the target domain caused by preceding learning processes in the source domain [33,40]. “Parameter efficiency” reduces the amount of computation in the target domain [41,42]. “Data efficiency” minimizes the amount of data required for adaptation in the target domain [30].

There are several related fields of research. “Domain generalization” tries to learn a predictor in a set of source domains so that it is invariant relative to distribution shifts. The predictor thus generalizes well in an unseen target domain [43,44]. “Few-shot learning” aims to learn ML models using only a small amount of training data for supervised training [45]. Research on “zero-shot learning” tries to train a model without supervised labeling in the target domain [46]. Recently, “prompt learning” from the field of natural language processing has been employed to attempt to reduce or avoid labeled data in the target domain. For this, it embeds the input into a prompting function and searches for the highest-scoring solution [47]. Some approaches transfer knowledge from a set of multiple neural networks in the source domain (“model zoo”) [48]. In “model selection,” different models are compared with respect to their suitability for a specific transfer learning problem [49]. “Continual learning” is a technique used for training neural networks with respect to multiple tasks in succession without unlearning previously learned tasks [50].

### 2.2. Task Adaptation Techniques

**Figure 2:**
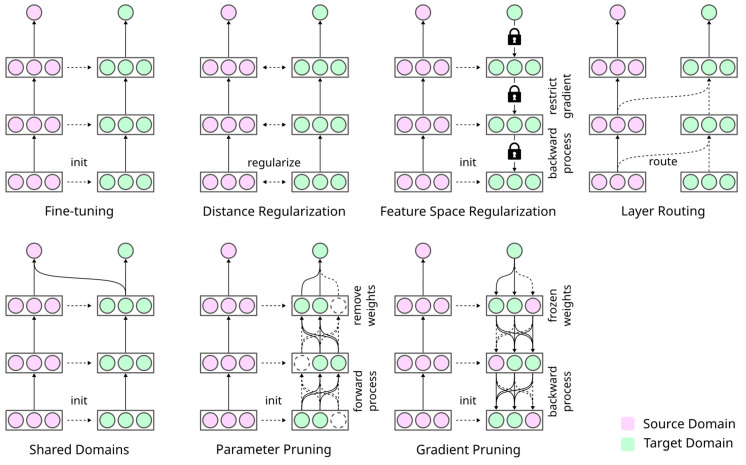
Technical perspective on different forms of task adaptation. Adapted with permission from Ding et al. [51] and extended by introducing the categories “feature space regularization” and “shared domains”.

From another perspective, task adaptation strategies can be broadly categorized into seven categories of technical approaches (see Figure 2) [51]. In the following, we describe task adaptation techniques from the literature relative to image classification (see Table 1). In each case, we classify the techniques in a technical scheme.

#### 2.2.1. Vanilla Fine-Tuning

Regular “vanilla” fine-tuning is relatively simple and usually includes three steps [7,20,21]. First, one trains a neural network on a source task. Second, the last layers of the network are usually replaced by newly initialized layers. Third, single layers are either frozen or adapted to a target task using additional supervised training.

#### 2.2.2. Distance Regularization

“Distance regularization” attempts to minimize the distance between fine-tuned weights and pre-trained weights [51]. It thereby preserves knowledge from the source domain. Most techniques rely on the loss function to regulate the feature space. “Explicit Inductive Bias” (L^2^-SP) [24] is based on the standard L^2^ regularization called “weight decay”. New layers that are added to the network and that are then reinitialized use this regularization. The procedure adds a second loss term, which penalizes large distances between the fine-tuned and pre-trained weights. The strength of both loss components is controlled using two parameters: *α* and *β*. “MARS-SP” [52] penalizes the maximum absolute row sum (MARS) distance between the weights of the source and the target domain. “DEep Learning Transfer using Feature Map with Attention” (DELTA) [53] is similar to L^2^-SP. However, it uses the distance between the feature maps of the source and the target model (instead of the layer weights). The distance is weighted by the performance reduction caused by turning off filters one at a time.

Instead of limiting the deviations using the loss function, hard constraints can be used. Gouk et al. [52] studied two variants. “L^2^-PGM” [52] uses a variant of the projected stochastic sub-gradient method with a constraint on the Frobenius distance. “MARS-PGM” [52] uses a constraint on the MARS distance instead.

**Table 1:** Overview of task adaptation techniques from the literature on image classification and categorized from a technical perspective.

Technique	Reference	Category	Ranking	Source Code
**Distance Regularization**				
DELTA	[53]	CF	A*	✓
L^2^-PGM	[52]	CF	A*	✓
L^2^-SP	[24]	CF	A*	✓
MARS-PGM	[52]	CF	A*	✓
MARS-SP	[52]	CF	A*	✓
**Feature Space Regularization**				
Bi-Tuning	[54]	DE	A	✓
BSS	[55]	NT	A*	✓
DTNH	[15]	NT	A*	✗
StochNorm	[56]	DE	A*	✓
**Layer Routing**				
AdaFilter	[57]	CF	A*	✗
DEFT	[58]	CF	Q1	✗
DKL	[59]	CF	Q2	✓
Flex-Tuning	[60]	CF	A	✗
MultiTune	[61]	CF	B	✓
PTU	[62]	CF	A	✗
SpotTune	[23]	CF	A*	✓
PathNet	[63]	CF	B	✗
Stepwise PathNet	[64]	CF	Q1	✗
**Shared Domains**				
Co-Tuning	[22]	CF	A*	✓
LwF	[65]	CF	Q1	✓
Selective Joint Fine-tuning	[66]	DE	A*	✓
**Parameter Pruning**				
Ticket Transfer	[67]	PE	A*	✓
Winning Lottery Tickets	[68]	PE	B	✓

Category descriptors include: catastrophic forgetting (CF), negative transfer (NT), parameter efficiency (PE), or data efficiency (DE). Ranking according to SCImago Journal Rank (SJR) indicator (Q1, Q2, Q3, or Q4) or CORE Conference Ranking score (A*, A, B, or C).

#### 2.2.3. Feature Space Regularization

The “feature space regularization” techniques apply some restrictions on backpropagation to ensure the desired properties of the fine-tuned feature space. Two techniques belong to the category of catastrophic forgetting. “Stochastic Normalization” (StochNorm) [56] replaces standard batch normalization with a two-branch version. One branch uses means and mini-batch variances; the other uses moving statistics. The branches are selected stochastically. In this manner, over-fitting is penalized more, and more knowledge is also transferred. “Bi-Tuning” [54] adds a newly designed categorical contrastive learning loss to the loss term to better make use of the intrinsic structure of pre-trained feature representations.

Two other methods try to reduce negative transfer. “Batch Spectral Shrinkage” (BSS) [55] applies singular value decomposition (SVD) on feature matrices. It assumes that spectral components with small singular values are not transferable. In order to suppress negative transfer, BSS penalizes the smallest singular values by using an additional loss term. Wan et al. [15] developed “Descent Direction Estimation Strategy” (DTNH), which evaluates the gradients of empirical loss and regularization separately. In the case of an obtuse angle between those two, it decomposes the regularization gradient into two orthogonal vectors. One of these two vectors, which is parallel to the empirical gradient, is then truncated. This prevents the empirical loss descent from slowing down.

#### 2.2.4. Layer Routing

For “layer routing,” some of the pre-trained layers are either frozen or disconnected [51]. The selection of layer subsets is carried out using different approaches. Routing can be performed using modifications of the model architecture itself. “MultiTune” [61] applies L^2^-SP on two parallel ResNet models with different parameters each. Then, a single fully connected layer called “MultiTune layer” is trained to combine the output of these two models. “Parameter Transfer Unit” (PTU) [62] uses two parallel neural networks. One is trained in the source domain and then frozen. The other is trained from scratch in the target domain. Between the layers, two neurons called “fine-tune gate” and “update gate” learn to combine the activations of both models.

Some methods identify layers for fine-tuning using simple algorithmic approaches: “Flex-tuning” [60] decomposes a neural network into subcomponents. It identifies one component that should be fine-tuned (while the others are frozen). The fastest variant, “even faster flex-tuning,” carries out a single fine-tuning epoch. Then, each fine-tuned block is copied into a separate network with pre-trained weights to obtain a proxy measurement of accuracy. After selecting the best block, it is fully fine-tuned. “DKL” [59] uses the Kullback–Leibler divergence on weight correlations to identify the best layers for fine-tuning.

Some techniques use an additional policy network for routing prediction: “SpotTune” [23] is based on the view that ResNet is an ensemble of shallow networks [69]. The approach uses two copies of the pre-trained model: a frozen and a trainable one. For each image, a policy network decides whether a pre-trained or fine-tuned block of ResNet should be used. As the binary policy vector itself is discrete and thus non-differentiable, the Gumbel–SoftMax trick is used during the backward pass. “AdaFilter” [57] uses pre-trained and fine-tuned filters. A binary vector called “fine-tuning policy” decides which filters to use on a per-image basis. These fine-tuning policies are predicted using a recurrent gated network. The network is trained using the “straight-through estimator” because of the binary values during the forward pass. Gated batch normalization replaces standard batch normalization to handle domain shifts.

Some approaches make use of evolutionary algorithms for routing: “PathNet” [63] finds binary fine-tuning genotypes (fine-tune/do not fine-tune specific layers) using a genetic algorithm. The best genotypes are combined into an ensemble. In “Stepwise PathNet” [64], a tournament selection algorithm based on a microbiological genetic algorithm carries out selections between frozen pre-trained and fine-tunable pre-trained layers. Similarly to PathNet, “Differential Evolution based Fine-Tuning” (DEFT) [58] uses an evolutionary algorithm (differential evolution) to select layers for fine-tuning. However, real-valued genotypes (instead of binary values) are used. For training, phenotypes are binarized using a simple threshold. In the end, a final model is fine-tuned based on the results.

#### 2.2.5. Shared Domains

The category “shared domains” uses information from the source domain for additional training supervision during the backpropagation step. “Selective Joint Fine-tuning” [66] searches for training images with visually similar low-level characteristics. Two output layers and two cost functions are used to simultaneously train the network on the source and target datasets. “Learning without Forgetting” (LwF) [65] uses the pre-trained classifier to predict labels for new images in the source label space. Then, new output layers are initially trained (warm-up step), while previous layers are kept frozen. Finally, with all layers unfrozen, the model is jointly trained to predict the pre-computed source labels and the new layers using a combined loss term. “Co-Tuning” [22] translates target labels into probability distributions in the source domain. During training, the translated probability distributions combined with the source classifier layers are used to add an additional element to the loss function.

#### 2.2.6. Parameter Pruning

“Parameter pruning” leverages the structural information of the pre-trained model from the source domain [51]. van Soelen and Sheppard [68] carried out training on a source dataset. Based on the “Winning Lottery Tickets hypothesis” [70], they identified winning tickets using one-shot pruning. The authors then retrained them on the target dataset. Each layer was pruned individually, and bias terms were excluded. Mehta [67] also adopted the procedure by Frankle and Carbin [70] and validated what they called the “Ticket Transfer Hypothesis”. However, they applied iterative pruning. The weights of the winning ticket were then transferred to the target domain.

## 3. Methodology

This section describes the methodology of our study with respect to task adaptation and its various techniques. As shown in Section 2, task adaptation comprises several closely related research areas as well as a variety of algorithms. Therefore, in this section, we begin with a precise delimitation of our object of investigation based on various criteria. Secondly, we describe an appropriate experimental design to answer our research question for the previously delimited algorithms. Thirdly, our research question itself does not imply a specific DL pipeline architecture. To collect empirical results, however, we must restrict ourselves to a single representative architecture. We therefore define and describe the exemplary DL CNN pipeline used in this study. Finally, we lay out the details regarding its hardware and the software implementation.

### 3.1. Transfer Learning Techniques

Our study defined a specific application scenario regarding the availability of labeled data: we assumed that there was a small amount of labeled data available. While the amount was enough for supervised model training, it was not sufficient for “training from scratch”. Therefore, we subsequently disregarded the application areas of data efficiency, domain adaptation, and the related areas of few-shot/zero-shot learning. Furthermore, we were interested in training a model with high performance. Thus, we were not interested in runtime performance and disregarded parameter efficiency as well. Our focus was, therefore, on task adaptation, especially catastrophic forgetting and negative transfer. We further narrowed down the use case scenario. We assumed that there was only one model in the source domain (no model zoo) and that the time-consuming pre-training was already carried out (no model selection). The focus was on the target domain; thus, accuracy in the source domain was irrelevant (no continual learning).

The task adaptation techniques to be included in our study were selected according to several criteria. One constraint was to keep the total computation time of our experimental setup feasible with respect to our available computational resources. Model training in DL takes significant time and resources. More specifically, we had to train *m* × *n* × *k* models, where *m* is the number of techniques to be evaluated, *n* is the number of tasks, and *k* is the number of training repetitions [71]. Another motivation was to restrict the findings to procedures that are relevant to practical applications.

For the reasons mentioned above, we defined the following theoretical and practical inclusion criteria:

- We required the code availability of a PyTorch/TensorFlow/Keras implementation.
- The task adaptation technique was designed for/was suitable to image classification models.
- The task adaptation technique was compatible with the ConvNeXt architecture.
- The task adaptation technique was designed for high accuracy/AUC (catastrophic forgetting and negative transfer).
- The original publication was published in a peer-reviewed journal/conference and holds a good ranking in the SJR (Q1) or CORE ranking (A*, A, B).

Furthermore, the following restrictions were used to reduce the computational complexity of our evaluation:

- We dropped L2-PGM and MARS-SP, as MARS-PGM seems to be favored by Gouk et al. [52].
- We dropped LwF in favor of Co-Tuning as the approaches are similar, and You et al. [22] found that “Co-Tuning fits better for transfer learning than LwF because Co-Tuning explicitly models the category relationship” [22].

We finally included DELTA, L^2^-SP, and MARS-PGM from distance regularization; Bi-Tuning and BSS from feature space regularization; MultiTune and SpotTune from layer routing; and Co-Tuning from shared domains. As a baseline, we chose to train a model using the standard vanilla fine-tuning approach [7].

### 3.2. Experimental Design

In our experimental design, we limited ourselves to a subset of histopathological classification tasks and their corresponding public datasets. Then, we distinguished between two aspects: comparing task adaptation algorithms and comparing training dataset sizes, each with respect to their achieved accuracy. The respective evaluation procedures follow the latest best practices for the comparison of ML algorithms to ensure generalizable findings [71,72].

#### 3.2.1. Classification Tasks and Datasets

The study focused on histopathology in general and therefore addressed exemplary clinical tasks based on previously published datasets. To cover a wider variety of application scenarios in histopathology, we included five classification tasks from multiple cancer types. We also collected multiple public image datasets for each task. The following describes the classification tasks and the corresponding datasets in detail.

*Mitotic Figure Detection.* Our first classification task was partly adopted from Tellez et al.’s study [36]. In this case, the task was to detect mitotic figures in the center of image patches from breast cancer. The predicted variable is binary, namely, “mitotic figure present” vs. “no mitotic figure present” in the center. As our first dataset, we used the “breast cancer histopathological annotation and diagnosis dataset” (BreCaHAD) [73]. Hematoxylin and eosin (H&E)-stained images with a size of 1360 × 1024 pixels were collected at the University of Calgary. Our second dataset comes from the “TUmor Proliferation Assessment Challenge” in 2016 (TUPAC16) [74]. More specifically, the “mitosis detection dataset” consists of H&E-stained square images of different sizes. It was gathered from three pathology centers in The Netherlands: the University Medical Center in Utrecht, the Symbiant Pathology Expert Center in Alkmaar, and the Symbiant Pathology Expert Center in Zaandam. The images from the TUPAC16 and the BreCaHAD datasets had a spatial resolution of 0.25 µm/pixel.

*Tumor Metastasis Detection.* The second classification task was partly adopted from Tellez et al.’s study [36] as well. The purpose of the classifier was to detect patches containing metastatic tumor cells of breast cancer. Therefore, the binary classes were “metastatic tumor cells present” and “others”. Both the training and test data came from the “CAncer MEtastases in LYmph nOdes challeNge” in 2017 (CAMELYON17) [75]. The annotated data included H&E-stained whole-slide images (WSIs) from the Radboud University Medical Center (Radboudumc), the Utrecht University Medical Center, the Rijnstate Hospital, the Canisius-Wilhelmina Hospital, and the LabPON in The Netherlands. Model training and external testing were performed on the data from the first, second, third, and fourth centers. Image data from all centers in the CAMELYON17 dataset had a spatial resolution of 0.23–0.25 μm/pixel.

*Tumor-infiltrating Lymphocytes (TIL) Detection.* The third task was to classify TIL-positive/-negative image patches. In this case, TIL-positive meant that at least two TILs were present in the image [76,77,78]. The dataset included H&E-stained square images with 100 × 100 pixels from 22 cancer types in “The Cancer Genome Atlas” (TCGA) program. We excluded four cancer types (CESC, LUSC, READ, and STAD) as they were annotated using DL. This resulted in a dataset of 18 remaining TCGA projects (ACC, BRCA, COAD, ESCA, HNSC, KIRC, LIHC, LUAD, MESO, OV, PAAD, PRAD, SARC, SKCM, TGCT, THYM, UCEC, and UVM). We obtained two independent, non-overlapping mitosis datasets by randomly dividing cancer types into two splits. The spatial resolution of all image patches in TCGA-TIL was 0.50 µm/pixel.

*Colorectal Cancer Tissue-type Classification.* Again, based on the study by Tellez et al. [36], the goal of colorectal cancer (CRC) tissue type classification was to distinguish among six different CRC tissue classes. The classes for classification were “tumor epithelium,” “simple stroma,” “immune cells,” “debris and mucus,” “normal mucosal glands,” and “adipose tissue”. As the first dataset, CRC-5000 by Kather et al. [79] was used. They provided 5000 patches (625 per class) of archived H&E-stained WSIs from the University Medical Center Mannheim. Each patch had a resolution of 150 × 150 pixels. As the second dataset, the colon cancer dataset (DRCO) from the DROID project was used [80]. DRCO provided H&E-stained WSIs with annotation masks. To align the classes for CRC-5000 and DRCO, we dropped the “complex stroma” and “background” classes from CRC-5000. The severe imbalance of some classes made it difficult to divide the classes into training and test splits without data leakage. Thus, we excluded two “immune cells” and “debris and mucus” classes for the task “DRCO Small”. The spatial resolution of CRC-5000 was 0.50 µm/pixel, and DRCO used a resolution of 0.45–0.50 µm/pixel.

*Skin Cancer Tissue-type Classification.* For skin cancer classification, the goal of the classifier was to distinguish among eight different skin tissue types. The tissue classes were “skin appendage” (including hair follicles and sweat glands), “inflammation,” “hypodermis,” “dermis,” “epidermis,” “basal cell carcinoma“ (BCC), “squamous cell carcinoma” (SCC), and “intraepidermal carcinoma” (IEC). We used a dataset that we subsequently referred to as “Queensland” as our first dataset. The dataset was published by Thomas et al. [81] and provided by MyLab Pathology in Australia. It included segmented H&E-stained WSIs of BCC, SCC, and IEC. As our second dataset, the skin cancer dataset (DRSK) from the DROID project was used [80]. DRSK provided H&E-stained WSIs with annotation masks. To align the classes of Queensland and DRSK, we dropped the “keratin” and “background” classes with respect to Queensland. Furthermore, “reticular dermis” and “papillary dermis” were combined into “dermis”. The classes “sweat glands” and “hair follicles” were combined into “skin appendage structure”. The severe imbalance of some classes made dividing these classes into training and test splits difficult without causing data leakage. Thus, we dropped three classes, BCC, SCC, and IEC, for the task “DRSK Small”. The spatial resolution of Queensland was 0.67 µm/pixel, while the images in DRSK had a resolution of 0.45–0.50 µm/pixel.

#### 3.2.2. Comparison of the Task Adaptation Techniques

The experimental design for comparing task adaptation techniques was based on the statistical comparison of multiple ML algorithms across multiple datasets, as described by Benavoli et al. [71]. Generally, for the comparison of *m* techniques on *n* tasks, model training is repeated *k* times for each combination of *m* and *n*. The mean values of the *k* repetitions form the set of observations per algorithm. These means are then statistically compared. For our case with *m* = 9 techniques, *n* = 12 tasks, and *k* = 5 repetitions, this resulted in a set of 9 × 12 × 5 = 540 models in total.

Our experimental design was robust. Multiple tasks were used to evaluate the classifier (see Table 2). We carried out stratified splitting with respect to the training and validation datasets at the case level to avoid patient-specific data leakage. For a more realistic task adaptation scenario, only a fraction of all data was randomly sampled for training and validation. The proportions were chosen so that the smaller class sizes were roughly in the lower three-digit range, similarly to other experiments on task adaptation [22,24,53]. However, achieving equal dataset sizes among the different tasks was challenging due to varying class imbalances. Training and subsequent testing were repeated five times. We kept the stratified random samples constant across the evaluated algorithms [72]. For every task, we tested them on independent external test datasets. For computational efficiency, a stratified random subset with a maximum size of 100,000 patches was chosen for all datasets.

The experimental design includes solid statistical validation. The area under the curve (AUC) was chosen as the final measure. It is a standard metric for comparing classification algorithms [72]. For each combination of tasks and algorithms, the mean and standard deviation of the AUC values were calculated and reported in the Results Section. We used statistical tests to verify the generalizability of our results beyond the tasks in our experimental setup [82]. The classical null hypothesis significance testing (NHST) procedure was recently criticized for being inappropriate for ML algorithm comparisons. We therefore used Bayesian hypothesis testing instead [71].

Bayesian testing is preferable because it avoids several shortcomings of classical NHST [71]. With respect to NHST, decisions are made based on the probability of observing an effect when the actual mean difference of two classifiers, *A* and *B*, is assumed to be 0 (*H*_0_ hypothesis). Bayesian testing, instead, can directly estimate the probability of *A* being better than *B* (and vice versa) based on some observations. Furthermore, NHST is based on the unrealistic assumption that two algorithms can potentially have equal performance. Thus, trivial effect sizes can become significant by increasing the number of observations. In Bayesian testing, one can define a region of practical equivalence (ROPE) to take this problem into account beforehand. NHST does not allow drawing conclusions from non-significant results. However, Bayesian testing can show the equivalence of two algorithms based on the ROPE.

In addition to Bayesian testing, we additionally studied effect sizes [83]. Given means *m_A_* and *m_B_* and the pooled standard deviation, *σ_pooled_*, of two normally distributed populations *A* and *B*, Cohen’s *d* is calculated as follows [84]:

(1) $$d=\frac{m_{A}-m_{B}}{\sigma_{pooled}}$$

We omitted effect sizes in the pairwise comparison based on multiple datasets in Section 4.1. In this case, the denominator in Equation (1) reflects the classification tasks’ difficulty and not the model’s performance dispersion. As a nonparametric variant of Cohen’s d for populations without a normal distribution, we adopted Akinshin’s gamma from the Python package “Autorank” [85].

#### 3.2.3. Comparison of Training Dataset Sizes

We were also interested in how the performance of the techniques under investigation would behave with a larger amount of data. The performance gain of vanilla fine-tuning is known to become saturated with the increase in the dataset size [8]. Some recent studies on advanced task adaptation techniques carried out comparisons with respect to the training datasets’ size. Some techniques performed better in a large-scale setting with more than 1000 images per class, while others did not [22,54,56]. To evaluate the impact of dataset sizes in histopathology, we chose to re-evaluate some of the task adaptation techniques using additional experiments.

The experimental design for comparing multiple dataset sizes was based on the statistical comparison of two algorithms using one dataset, as described by Benavoli et al. [71]. In this case, we compared an algorithm trained on a small training split vs. trained on a larger training split of the same dataset. Therefore, for each combination of *m* techniques and *n* training splits, *k* repetitions of the model training were performed. We chose three techniques (L^2^-SP, fine-tuning, and Co-Tuning) from our set of task adaptation algorithms. We re-used dataset #10 from the colorectal cancer tissue-type classification (see Table 2). Three variants of the training split were generated using a varying subsampling factor: “Base,” “Large,” and “XLarge” (see Table 3). Base is equivalent to the size shown in Table 2. For every combination of split size and learning technique, we trained *k* = 20 models. With *m* = 3 techniques, *n* = 3 training split variants, and *k* = 20 repetitions, this resulted in a set of 3 × 3 × 20 = 180 models in total. Again, the AUC was chosen as our metric for comparisons. We verified the results using the Bayesian correlated *t*-test. We further report effect sizes in the Results Section.

### 3.3. Exemplary Image Classification Pipeline

A DL pipeline for image classification typically includes multiple sequential components. Each component comes with its own set of design choices. Here, we limited ourselves to a single exemplary DL pipeline, used for all experiments in this study. The core building blocks are typically image preprocessing and the neural network itself. We first started with the definition of our image preprocessing. We then described the state-of-the-art CNN architecture. Finally, we addressed the hyperparameters of our pipeline.

#### 3.3.1. Image Preprocessing

Neural networks for image processing typically operate on smaller image patches. Therefore, we used square image patches with a default edge length of 128 pixels. The spatial resolution of the training and testing images were identical for all tasks, except for the skin cancer tissue-type classification. To account for the different spatial resolutions in the latter case, we used patch sizes with an edge length of 128 × (0.67/0.5) = 171 pixels for the DRSK dataset. Subsequently, all patches were scaled uniformly to 224 × 224 pixels, the default input image size of ConvNeXt.

Inequalities in class distribution must be considered when training representation learning algorithms. An imbalance of varying magnitudes is present in all datasets, except for CRC5000. Oversampled minority classes were therefore used during training time. This prevents the classifier from becoming biased towards one or more specific class(es). For validation and testing, we kept the original class distribution of the datasets.

When training neural networks for image processing, increasing the training set size using various image augmentations is common. We used random horizontal flipping. Following the advice of Tellez et al. [36], we further used HSV color augmentation. As commonly carried out in PyTorch, the mean and standard deviation were normalized to [0.485, 0.456, 0.406] and [0.229, 0.224, 0.225] in the RGB color space, respectively.

#### 3.3.2. CNN Architecture

Since the development of AlexNet in 2012 [86], neural networks based on convolution have been widely used as a standard approach for CV. Research has constantly improved network architecture around this fundamental principle, including architectural milestones like VGG [87], ResNet [88], Xception [89], or EfficientNet [90]. Recently, transformer-based architectures from natural language processing (NLP) challenged the use of convolution operations [91,92,93,94]. Other than CNNs, they are based on multi-head self-attention.

Whether to use convolution- or attention-based architectures for CV remains an open research question. Liu et al. [95] found that transformers gain importance compared to CNNs mainly because of their better scaling behavior. However, recent studies showed that CNNs can still achieve an equivalent or better performance than transformers after optimizing several design choices. One of these options is larger kernel sizes [95,96,97,98,99]. This raises the question of whether the recent performance of transformer models is really a result of multi-head self-attention. At the same time, the established convolution approach was designed around efficiency [95]. The new design principle of choosing large kernels also seems to push the networks to shift towards a shape bias [97]. This effect resolves a previous issue of pre-trained CNNs being biased due to texture [100].

**Figure 3:**
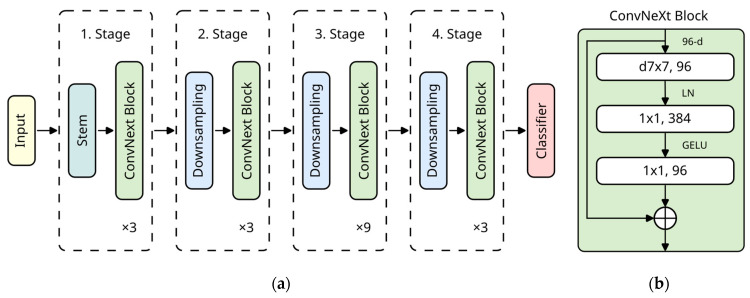
(**a**) Overall architecture of ConvNeXt Tiny [95]. The macro-level architecture with a ratio of 1:1:3:1 was adopted from Swin Transformers [103]. (**b**) Detailed structure of the ConvNeXt block [95]. The micro-level architecture is based on the inverted bottleneck design [104]. Inspired by transformer architectures, the depthwise convolution uses a kernel size of 7 × 7. For computational efficiency, it is computed before the expansion of channels. The Gaussian error linear unit (GELU) [105] and layer normalization [106] were also adopted from recent transformer-based architectures [95]. Adapted from Jiang et al. [107], © 2023, Frontiers Media S.A., CC BY 4.0 DEED.

For this study, we chose a state-of-the-art CNN architecture. As described above, the newest architectures achieved cutting-edge performance for image classification. Furthermore, most task adaptation techniques were explicitly designed for CNNs. Therefore, we selected ConvNeXt [95] to represent novel CNN architectures. We chose the smallest one, “ConvNeXt Tiny”, as we assumed it had enough capacity for our tasks while keeping training time manageable. The building blocks are shown in Figure 3. The pre-trained ImageNet weights originate from PyTorch [101]. We transferred these weights to all used models. Furthermore, we ensured that all implementations (both in PyTorch and Keras) produced the same model results (excluding rounding errors) so that different model implementations do not distort the results.

#### 3.3.3. Hyperparameters

For our experiments, we preferred the stochastic gradient descent (SGD) optimizer over Adam, another common choice, based on two reasons. First, SGD generalizes better for image classification [102]. The performance of Adam seems to depend on the hyperparameter optimization of decay rates *β*_1_ and *β*_2_. Second, Adam was only used in the study performed by Gouk et al. [52]. The authors of the other studies used SGD. From a practical perspective, this results in less implementation effort when using SGD as well.

Broadly consistent parameters for SGD were used across the algorithms. In line with recent recommendations on fine-tuning in dissimilar source and target domains [17], we used SGD with an initial learning rate of 0.01 and a momentum of 0.9 and activated Nesterov. We adopted step decay from the majority of the evaluated studies [7,23,24,52,53,54,61]. We consistently set a milestone at the 10th epoch with a gamma value of 0.1. We used a lower learning rate for the pre-trained layers in the fine-tuning setting. We also adopted lower learning rates for the policy network of SpotTune and the last block in MultiTune from the original studies [23,61]. To avoid exploding gradients, we applied gradient value clipping with a value of 1.0. We chose to use a batch size of 64. During training, iterations per epoch were fixed to 128. Models were trained with early stopping, patience of 15, and a maximum number of 100 epochs.

Various hyperparameters must be specified for the algorithms under study. However, conducting hyperparameter optimization to identify optimal parameters for each task on each dataset was not practical due to the substantial computation time required. Instead, we utilized the suggested values from the literature. Li et al. [108] recently found that L^2^-SP is robust across different parameter ranges. However, *α* > *β* is generally preferable. Therefore, we extracted *α* = 0.1 and *β* = 0.01 from the original study [24]. For MARS-PGM, we used the average values of *γ_j_* = 9.32 and *γ_L_* = 16.89 [52]. For DELTA, Li et al. [53] fixed *β* = 0.01 and generally used it for all conditions [53]. We further set *α* = 0.04. For Co-Tuning, You et al. [22] found that *λ* = 2.3 is robust across all tested datasets and sampling rates. For BSS, Chen et al. [55] found that *η* = 0.001 and *k* = 1 are generally adequate settings under varying conditions. Zhong et al. [54] universally applied *τ* = 0.07 and *m* = 0.999 for Bi-Tuning. For a 100% sampling rate, which is comparable to our class size, they found that a high number of sampling keys, *K*, is beneficial. In line with this, we therefore used the following defaults: *K* = 40 and *L* = 128. Wang et al. [61] used *α* = 0.01 and *β* = 0.01 for all their experiments.

For our baseline, vanilla fine-tuning, the model weights were copied from the ImageNet domain. The pre-trained classification head was replaced by a new one with randomly initialized weights. The new model was then fine-tuned on the histopathology tasks. For the pre-trained feature extractor, a smaller learning rate (10 times smaller) was chosen.

### 3.4. Hardware and Software Implementation

Our server had two AMD EPYC 7402 24-core processors, one terabyte of random-access memory, NVIDIA RTX A6000 graphics cards, and an Ubuntu 22.04 LTS operating system. Various freely available software packages were used. All experiments were implemented in Python (ver. 3.9.16). WSI data were loaded using the Python package tiffslide (ver. 2.1.2) [109]. Model training was implemented using PyTorch (ver. 2.0.0) [110], Keras (ver. 2.11.0), and TensorFlow (ver. 2.11.1) [111] libraries. For PyTorch training, image augmentations used the Transforms module from Torchvision (ver. 0.15.1). For Keras training, the same augmentations were implemented using the Albumentations (ver. 1.3.0) package [112]. For the calculation of general sample statistics and the Bayesian signed-rank test, we used the implementation of the Python package Autorank (ver. 1.2.0) [85]. The Bayesian correlated *t*-test was calculated using baycomp (ver. 1.0.3) [113].

## 4. Results

In the following, the experimental results are presented. As defined in Section 3.2.2, we begin by comparing different task adaptation techniques. We then compare the accuracy of several algorithms for different training data sizes, as described in Section 3.2.3.

### 4.1. Comparison of the Task Adaptation Techniques

As described in Section 3.2.2, we trained 540 models for our first experiment on task adaptation techniques across different tasks. Table 4 shows the aggregated results. Table 5 shows some general statistics (median, median absolute deviation from the median, and confidence interval for the median) of the results. The statistics were ranked using the median AUC in descending order. Furthermore, the effect size was provided for the difference from the best algorithm using Akinshin’s gamma. Based on these results, we carried out a Bayesian signed-rank test for nine algorithms with paired samples (*n* = 12) and significance levels of *P*(·) ≥ 0.9, 0.95, and 0.99. Using the Shapiro–Wilk test for normality with Bonferroni correction, Autorank failed to reject the null hypothesis of normal distributions for MARS-PGM (*p* = 0.002). Therefore, a normal distribution of the results was not given for all algorithms. Based on the recommendations of Kruschke and Liddell [114], Autorank used 0.1 × MAD for the region of practical equivalence (ROPE) around the median.

Autorank reported significant and practically relevant differences between the algorithms L^2^-SP (MD = 0.930 ± 0.096; MAD = 0.021), fine-tuning (MD = 0.911 ± 0.105; MAD = 0.042), Co-Tuning (MD = 0.898 ± 0.082; MAD = 0.038), DELTA (MD = 0.893 ± 0.136; MAD = 0.040), SpotTune (MD = 0.889 ± 0.093; MAD = 0.020), BSS (MD = 0.881 ± 0.100; MAD = 0.044), Bi-Tuning (MD = 0.878 ± 0.086; MAD = 0.033), MultiTune (MD = 0.849 ± 0.086; MAD = 0.051), and MARS-PGM (MD = 0.802 ± 0.215; MAD = 0.037). Compared to the top-ranking algorithm, L^2^-SP, the mean difference was inconclusive for fine-tuning and Co-Tuning. DELTA was significantly smaller (*P*(≪) = 0.949) than L^2^-SP with a medium magnitude. There was a significant difference with a large magnitude for SpotTune (*P*(≪) = 0.962), Bi-Tuning (*P*(≪) = 0.934), MultiTune (*P*(≪) = 1.0), and MARS-PGM (*P*(≪) = 1.0).

**Figure A1:**
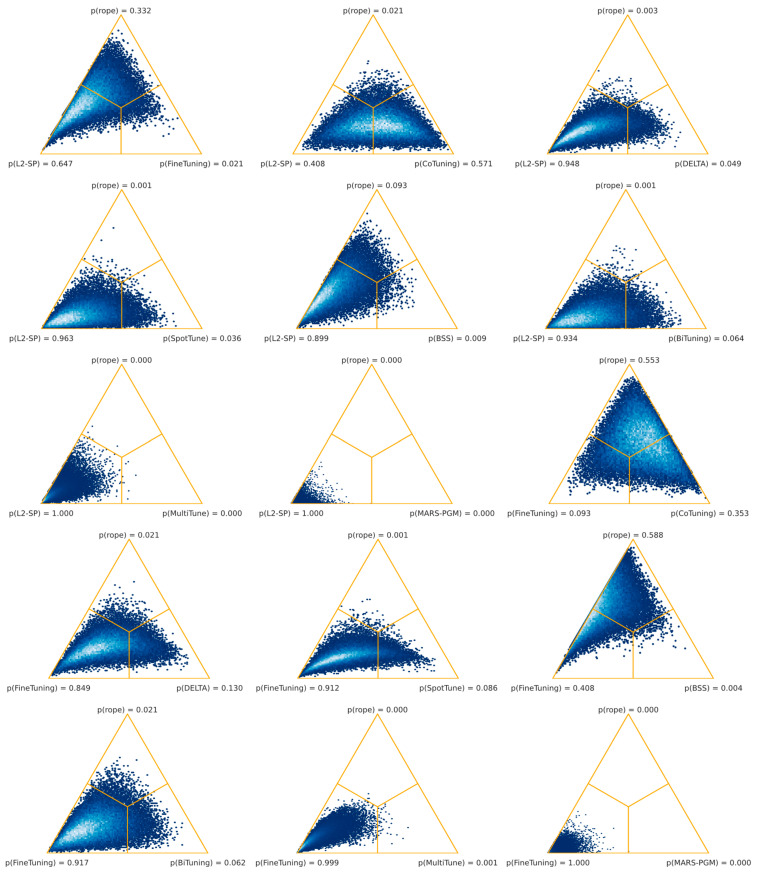

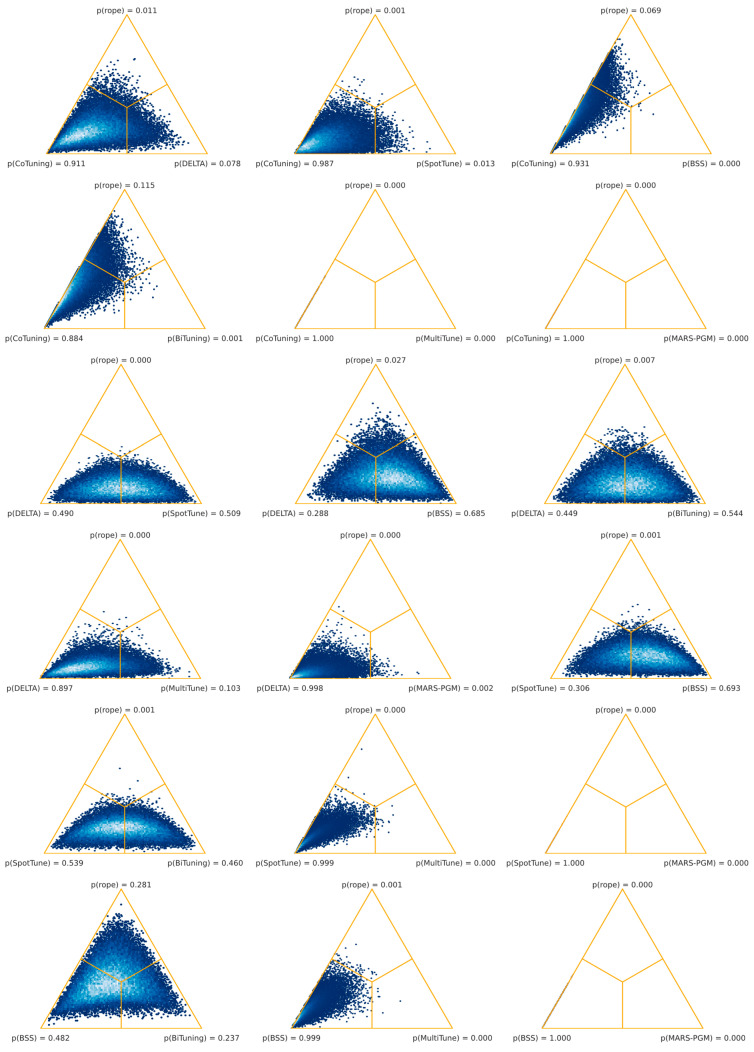

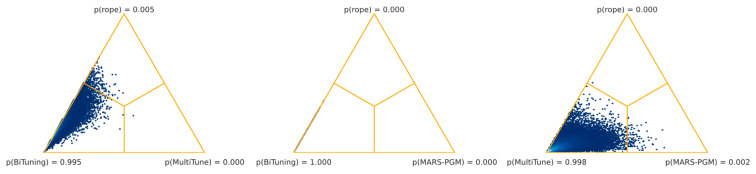
Simplex plots for the Monte Carlo sampling results of the pair-wise Bayesian signed-rank test.

Table 6 shows the results of the Bayesian signed-rank test for all possible pair-wise comparisons. Figure A1 in Appendix A shows the simplex plots from Monte Carlo sampling. Table A1 in Appendix B lists the exact aggregated probability values for all comparisons. The pair-wise mean differences were inconclusive for the first three algorithms (L^2^-SP, fine-tuning, and Co-Tuning). With some exceptions, at least one of these algorithms achieved significantly better results than the following two-thirds. This first group was followed by a second group of algorithms (DELTA, SpotTune, BSS, and Bi-Tuning) with inconclusive pair-wise differences. Finally, the remaining two algorithms (MultiTune and SpotTune) showed significant negative mean differences in almost all cases. At this point, we emphasize that inconclusive results do not necessarily constitute evidence of equal performance.

While our baseline (fine-tuning) performed comparatively well overall, it only ranked 3.83 on average across all datasets with respect to the descriptive statistics shown in Table 4. The question is whether it is still the best choice for all histopathological tasks. To better understand the influencing factors, we compared it with all other task adaptation techniques at the task level using the Bayesian correlated *t*-test. The aggregated results are shown in Table 7. The exact probabilities and effect sizes are also listed in Appendix C. For 5 of the 12 tasks, significantly better results than vanilla fine-tuning with medium or large effects were obtained by one or more task adaptation techniques. Co-Tuning and DELTA were the most frequently represented here, with three significant results each. SpotTune was significantly superior in two cases. Both Bi-Tuning and L^2^-SP achieved significantly better results than using the fine-tuning process once. In Table A2, one can further find a large positive effect (*P*(≫) = 0.896, *γ* = 1.692) for DELTA and a medium positive effect (*P*(≫) = 0.890, *d* = 0.626) for L^2^-SP. In both cases, Bayesian testing narrowly missed the threshold for automatic decisions. Thus, none of the algorithms consistently performed better than the baseline across histopathology. However, the indifference in results is partly explained by the differences in classification tasks. The positive findings include both binary and multiclass classification tasks.

### 4.2. Comparison of Training Dataset Size

As described in Section 3.2.3, we trained 180 models for the comparison of the dataset’s size. The aggregated training results based on AUC, balanced accuracy, and F1 score for different dataset sizes are presented in Table 8, Table 9 and Table 10, respectively. For the dataset size XLarge, Co-Tuning achieved the highest absolute increase in performance in terms of AUC (+0.013), balanced accuracy (+0.045), and F1 score (+0.048).

**Figure A2:**
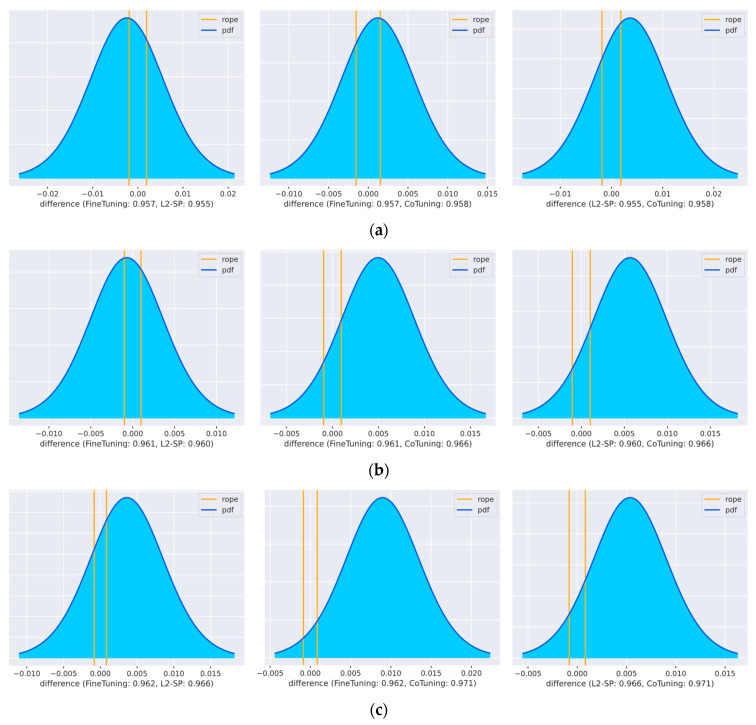
Posterior distributions for the pair-wise comparison of fine-tuning, L^2^-SP, and Co-Tuning using the AUC from the Bayesian correlated *t*-test. (**a**) Dataset size “Base” (×1). (**b**) Dataset size “Large” (×2.5). (**c**) Dataset size “XLarge” (×10).

Using the Bayesian correlated *t*-test, we carried out pair-wise comparisons between the algorithms for all three dataset sizes. Tables 11, 13 and 15 show descriptive sample statistics and Tables 12, 14 and 16 show the decision matrices for the settings Base, Large, and XLarge, respectively. The posterior distributions and probability values are presented in Figure A2 and Table A3–A5, respectively.

We started with the dataset size “Base”. According to the Shapiro–Wilk test for normality, the results follow a normal distribution. Therefore, we used parametric statistics to describe the samples (Table 11). The mean differences between Co-Tuning and the other two techniques were negligible in magnitude. For Bayesian testing, we used 0.1 × STD for the ROPE. Similarly to our experiments in Section 4.1, Bayesian testing produced inconclusive results (Table 12).

We continued with the dataset size “Large”. According to the Shapiro–Wilk test for normality, the results follow a normal distribution. Therefore, we used parametric statistics to describe the samples (Table 13). There were medium differences between Co-Tuning and the other two algorithms. For Bayesian testing, we used 0.1 × STD for the ROPE. The differences mentioned above were inconclusive in Bayesian testing (Table 14).

We further continued with the dataset size “XLarge”. According to the Shapiro–Wilk test for normality, the results do not follow a normal distribution. Therefore, we used nonparametric statistics to describe the samples (Table 15). For Bayesian testing, we used 0.1 × MAD for the ROPE. There was a significant medium increase (*P*(≫) = 0.951, *γ* = 0.748) for Co-Tuning over vanilla fine-tuning (Table 16). Overall, the results of the Bayesian testing across all three settings of our comparison of the datasets’ size thus confirm the increase in accuracy for the size XLarge.

## 5. Discussion

To the best of our knowledge, we are the first to provide extensive empirical evidence on advanced task adaptation techniques in histopathology. We showed that specific techniques are, on average, better suited for histopathology than others. Interestingly, the authors of all methods investigated in this paper observed, in their original studies, an improvement of their method over the baseline fine-tuning technique [23,52,54]. However, as shown in Table 6, this is not confirmed by our results for the histopathology setting. Vanilla fine-tuning significantly outperformed SpotTune, Bi-Tuning, MultiTune, and MARS-PGM. Three task adaptation techniques combined, namely, L^2^-SP, vanilla fine-tuning, and Co-Tuning, out-performed the others regarding classification tasks in digital histopathology. Among the three top-performing algorithms were distance regularization (L^2^-SP), shared domains (Co-Tuning), and the baseline technique (fine-tuning). Moreover, the worst-performing algorithms (MultiTune and MARS-PGM) were significantly outperformed by almost all other techniques, whereas MARS-PGM was significantly worse than MultiTune.

However, our results reveal that the superiority of an algorithm in histopathology depends on the task. Our systematic comparison, summarized in Table 6, did not show a general superiority of one task adaptation technique over the others. Also, none of the evaluated algorithms showed performances that were significantly superior to our baseline. However, the comparison between the three highest-ranking algorithms (L^2^-SP, fine-tuning, and Co-Tuning) was inconclusive as well. At first glance, this might be either due to our experimental setup, or performances might further depend on other influencing factors. Zhang et al. [33] already mentioned that, in addition to domain divergence and source/target data quality, the fit between the TL algorithm and the task is essential. Our task-level analysis confirmed this for histopathology (see Table 7). At least one technique obtained significantly better results than the baseline in about half of the cases.

Advanced task adaptation algorithms can also be helpful when a large amount of data are available. Our comparison of the dataset’s size showed that, by increasing the dataset size by a factor of ten, Co-Tuning was able to outperform the baseline technique significantly. This is in line with the results of You et al. [22]. They found that additional supervision by Co-Tuning helps to further increase performances in large-scale classification settings where regularization techniques, like L^2^-SP, do not help. Two other methods, which we did not examine in this large-scale setting, reported related results: the study on Bi-Tuning also reported a superior performance [54], while the study on StochNorm [56] obtained mixed findings.

The architecture of a neural network might be an influencing factor for the effectiveness of task adaptation. By choosing ConvNeXt, our investigation was based on an up-to-date CNN architecture from the field of CV. In general, ImageNet performance is a good indicator of the accuracy of CNN architectures relative to a downstream task [8]. However, Ding et al. [97] recently showed that CNNs with larger kernel sizes (like ConvNeXt) have higher shape biases compared to texture biases. In general research on CV, this is generally seen as beneficial due to its similarity with human perception [97,100,115]. While this might be true for photograph-like image data, histopathological analysis at a high magnification relies on relatively small cell structures and the visual texture of the tissue for decisions.

The algorithm-specific influence of histopathology pre-training needs to be further clarified. Image features from ImageNet and histopathology are likely to be different, and domain divergence is assumed to affect the result negatively [33]. For vanilla fine-tuning, three studies found that histopathology benefits from domain-specific pre-training [34,35,116], while two did not [117,118]. The question arises with respect to how advanced task adaptation techniques benefit from domain-specific pretraining and whether this effect diverges between algorithms.

Our results are in line with other medical studies. Several authors already provided evidence for the usefulness of advanced task adaptation techniques in a broader medical context. Most examples can be found in radiology. Liao et al. [25] used L^2^-SP to avoid catastrophic forgetting in a multitask setting called “MUSCLE”. They found that L^2^-SP was beneficial in all tested use cases. Sagie et al. [26] evaluated L^2^-SP in a supervised task adaptation setting for segmentation. L^2^-SP was not beneficial for ImageNet pre-training. However, it improved performances with respect to medical pre-training. Su et al. [27] successfully used L^2^-SP to carry out the supervised adaptation of an MRI-pre-trained GAN relative to CT image generation. Unfortunately, they did not compare their results to vanilla fine-tuning. An et al. [28] evaluated SpotTune in a supervised task adaptation setting for segmentation, which was pre-trained on a related medical dataset. SpotTune performed better than vanilla fine-tuning. Another example can be found in lung sound classification. Nguyen and Pernkopf [29] evaluated Co-Tuning, Stochastic Normalization, and a combination of both for supervised task adaptation with ImageNet pre-training. While Co-Tuning and StochNorm were superior to vanilla fine-tuning, their performance varied between classification tasks.

Our results add empirical evidence to CV research for the general relationship between domain divergence and negative transfer. The findings are based on eight techniques applied to the medical domain. We showed that the relative effectiveness of task adaptation techniques in histopathology diverges from general CV research. This observation confirms the assumption that performance depends on the domain [33]. Our findings might have implications for evaluating new transfer learning techniques. Increasing the variety in benchmark tasks has been proposed before [14,19]. In this paper, we confirmed that the evaluation of commonly used datasets containing animals, cars, aircraft, plants, etc., is insufficient.

However, the findings on some techniques also deviate from earlier findings in CV research in some ways. First, Li et al. [17] reported that distance regularization does not perform well for dissimilar source and target domains. Plested et al. [16] thus suggested that distance regularization should be limited to earlier layers, and the number of layers depends on the downstream dataset. Surprisingly, regular L^2^-SP distance regularization performed comparatively well in histopathology. Second, our results also contradict the conclusion of Gouk et al. [52], who reported that hard constraints perform better than distance constraints using the penalty term. MARS-PGM performed the worst of the algorithms compared (see Table 6).

## 6. Conclusions

This study is the first to provide empirical evidence on the suitability of up-to-date task adaptation techniques for histopathological classification tasks using CNNs. Analysis at the task level showed that, in half of the cases, one or multiple techniques, including Bi-Tuning, Co-Tuning, DELTA, L^2^-SP, and SpotTune, could outperform vanilla fine-tuning, which is the standard procedure for task adaptation. Co-Tuning can further increase performance when a large amount of data in the target domain are available. Our results align with earlier studies mainly from the radiological field, showing that medical AI can benefit from advanced task adaptation techniques. We further provided evidence on the general relationship between domain divergence and negative transfer. In this paper, we found some deviations from the literature on distance regularization.

### 6.1. Limitations

Our study was limited to supervised task adaptation techniques and excluded related research areas, like domain adaptation and zero-, one-, and few-shot learning. As we narrowed down the evaluated task adaptation techniques to the most promising ones in Section 3.1 for theoretical and practical reasons, our study did not cover 100% of all available methods from the literature. Furthermore, we limited our study to techniques that were initially designed for image classification. Our experiments did not combine BSS with distance regularization using L^2^-SP or DELTA. Although the classification tasks in Section 3.2 were based on 12 different datasets covering various cancer types and classification tasks, not all possible scenarios from histopathology were fully covered.

While we demonstrated relative improvements over the baseline approach (fine-tuning) for several tasks, our results do not show the best possible performance improvement due to the substantial computational costs required in our experimental setup for extensive hyperparameter optimization. We did not further investigate performances under varying influencing factors beyond dataset size—like different source domains, unsupervised pre-training, and the network’s architecture and size—or other downstream ML tasks—like instance segmentation. In our experiments, the number of layers using distance regularization was not adjusted relative to downstream tasks.

### 6.2. Outlook

This relationship between the histopathological task and the suitability of task adaptation techniques needs to be better understood in the future for task adaptation to be used in a targeted manner. More generally, the differentiated evaluation of transfer learning algorithms should continue so that more domains beyond ImageNet-like photographs can benefit more from task adaptation. Future studies should examine the impact of pre-training in pathology on task adaptation outcomes. More techniques, including Bi-Tuning [54] and StochNorm [56], should be investigated concerning their behavior in a large-scale setting.

## Figures and Tables

**Table 2:** Number of image patches per class for the training, validation, and test datasets. Training and validation data were split at the case level, while an external dataset was used for testing. Training and validation splits were extracted from the same image dataset.

Task	No.	Classes	Train	Validation	Test
Mitotic figure detection	#1		**TUPAC16**	**“**	**BreCaHAD**
		no mitosis	14,742	14,726	12,631
		mitosis	100	100	115
	#2		**BreCaHAD**	**“**	**TUPAC16**
		no mitosis	5042	6041	99,326
		mitosis	46	55	674
Tumor metastasis detection	#3		**Camelyon17, center 1**	**“**	**Camelyon17, center 2**
		no metastasis	5040	5400	99,421
		metastasis	100	100	579
	#4		**Camelyon17, center 2**	**“**	**Camelyon17, center 1**
		no metastasis	17,151	17,152	98,055
		metastasis	100	100	1945
	#5		**Camelyon17, center 3**	**“**	**Camelyon17, center 4**
		no metastasis	9473	9372	96,888
		metastasis	100	100	3112
	#6		**Camelyon17, center 4**	**“**	**Camelyon17, center 3**
		no metastasis	3112	3113	98,945
		metastasis	100	100	1055
Tumor-infiltrating lymphocyte detection	#7		**TCGA TILs, center 1**	**“**	**TCGA TILs, center 2**
		TIL-negative	656	656	73,687
		TIL-positive	100	100	18,030
	#8		**TCGA TILs, center 2**	**“**	**TCGA TILs, center 1**
		TIL-negative	408	408	86,778
		TIL-positive	100	100	13,222
Colorectal cancer tissue-type classification	#9		**CRC5000**	**“**	**DRCO**
		tumor	100	100	29,201
		stroma	100	100	32,166
		lympho	100	100	175
		debris	100	100	193
		mucosa	100	100	9565
		adipose	100	100	28,700
	#10		**DRCO Small**	**“**	**CRC5000 Small**
		tumor	305	305	625
		stroma	336	336	625
		mucosa	100	100	625
		adipose	300	300	625
Skin cancer tissue-type classification	#11		**Queensland**	**“**	**DRSK**
		SAS	56	56	5970
		INF	331	331	16,523
		HYP	1735	1735	41,060
		DER	4011	4010	30,845
		EPI	5	5	499
		BCC	149	149	3867
		SCC	416	416	1097
		IEC	100	100	139
	#12		**DRSK Small**	**“**	**Queensland Small**
		SAS	100	100	928
		INF	276	276	5403
		HYP	687	687	28,264
		DER	516	516	65,311
		EPI	8	8	94

TIL: tumor-infiltrating lymphocyte; SAS: skin appendage structure; INF: inflammation; HYP: hypodermis; DER: dermis; EPI: epidermis; BCC: basal cell carcinoma; SCC: squamous cell carcinoma; IEC: intraepidermal carcinoma.

**Table 3:** Number of image patches per class for three different dataset sizes. The factor of the dataset size is provided in brackets.

Classes	Base (×1)		Large (×2.5)		XLarge (×10)	
	Train	Validation	Train	Validation	Train	Validation
tumor	305	305	763	763	3052	3052
stroma	336	336	840	840	3362	3362
mucosa	100	100	250	250	1000	1000
adipose	300	300	750	750	3000	3000

**Table 4:** Mean and standard deviation of AUC values and per-task rank (in parentheses) for different task adaptation procedures.

Task	Baseline	Distance Regularization			Feature Space Regularization		Layer Routing		Shared Domains
	Fine-Tuning	DELTA	L^2^-SP	MARS-PGM	Bi-Tuning	BSS	MultiTune	SpotTune	Co-Tuning
#1	0.954 ± 0.014 (1)	0.926 ± 0.020 (5)	0.952 ± 0.014 (3)	0.780 ± 0.035 (9)	0.918 ± 0.023 (6)	0.939 ± 0.012 (4)	0.811 ± 0.044 (8)	0.901 ± 0.020 (7)	0.954 ± 0.019 (2)
#2	0.937 ± 0.028 (3)	0.676 ± 0.130 (9)	0.941 ± 0.016 (2)	0.770 ± 0.129 (8)	0.878 ± 0.071 (6)	0.919 ± 0.030 (4)	0.784 ± 0.139 (7)	0.887 ± 0.033 (5)	0.948 ± 0.009 (1)
#3	0.903 ± 0.029 (3)	0.897 ± 0.061 (4)	0.926 ± 0.023 (1)	0.763 ± 0.125 (9)	0.849 ± 0.031 (8)	0.893 ± 0.024 (5)	0.853 ± 0.044 (7)	0.866 ± 0.021 (6)	0.910 ± 0.034 (2)
#4	0.919 ± 0.024 (3)	0.943 ± 0.014 (1)	0.934 ± 0.019 (2)	0.804 ± 0.090 (8)	0.882 ± 0.049 (6)	0.832 ± 0.044 (7)	0.767 ± 0.107 (9)	0.906 ± 0.021 (4)	0.886 ± 0.035 (5)
#5	0.949 ± 0.010 (2)	0.948 ± 0.009 (3)	0.949 ± 0.009 (1)	0.837 ± 0.075 (9)	0.932 ± 0.007 (6)	0.940 ± 0.012 (4)	0.913 ± 0.014 (7)	0.891 ± 0.070 (8)	0.936 ± 0.010 (5)
#6	0.861 ± 0.051 (5)	0.848 ± 0.073 (8)	0.890 ± 0.042 (2)	0.473 ± 0.442 (9)	0.865 ± 0.050 (3)	0.864 ± 0.055 (4)	0.853 ± 0.036 (7)	0.890 ± 0.035 (1)	0.855 ± 0.076 (6)
#7	0.937 ± 0.007 (2)	0.899 ± 0.049 (9)	0.934 ± 0.012 (4)	0.902 ± 0.025 (8)	0.934 ± 0.012 (5)	0.938 ± 0.007 (1)	0.915 ± 0.015 (7)	0.916 ± 0.008 (6)	0.936 ± 0.010 (3)
#8	0.870 ± 0.023 (3)	0.888 ± 0.018 (1)	0.861 ± 0.024 (6)	0.800 ± 0.086 (9)	0.874 ± 0.017 (2)	0.870 ± 0.026 (4)	0.818 ± 0.117 (8)	0.849 ± 0.028 (7)	0.867 ± 0.021 (5)
#9	0.865 ± 0.011 (7)	0.874 ± 0.007 (3)	0.868 ± 0.011 (4)	0.831 ± 0.022 (9)	0.879 ± 0.008 (1)	0.866 ± 0.012 (5)	0.848 ± 0.019 (8)	0.865 ± 0.006 (6)	0.877 ± 0.005 (2)
#10	0.960 ± 0.009 (1)	0.928 ± 0.046 (8)	0.942 ± 0.032 (6)	0.856 ± 0.110 (9)	0.948 ± 0.028 (5)	0.953 ± 0.023 (4)	0.939 ± 0.025 (7)	0.959 ± 0.021 (2)	0.957 ± 0.015 (3)
#11	0.751 ± 0.036 (8)	0.793 ± 0.048 (1)	0.761 ± 0.037 (6)	0.737 ± 0.085 (9)	0.775 ± 0.011 (3)	0.753 ± 0.017 (7)	0.766 ± 0.010 (5)	0.773 ± 0.030 (4)	0.793 ± 0.022 (2)
#12	0.826 ± 0.020 (8)	0.808 ± 0.056 (9)	0.836 ± 0.036 (7)	0.845 ± 0.023 (4)	0.842 ± 0.079 (5)	0.842 ± 0.038 (6)	0.851 ± 0.048 (3)	0.875 ± 0.033 (1)	0.872 ± 0.015 (2)
Median (Avg.)	0.911 (3.83)	0.893 (5.08)	0.930 (3.67)	0.802 (8.33)	0.878 (4.67)	0.881 (4.58)	0.849 (6.92)	0.889 (4.75)	0.898 (3.17)

**Table 5:** Descriptive statistics for the ranked algorithms.

Algorithm	Median	MAD	CI	*γ*	Magnitude	*P*(≪)	*P*(=)
L^2^-SP	0.930	0.021	[0.761, 0.952]	–	–	–	–
Fine-Tuning	0.911	0.042	[0.751, 0.960]	0.391	small	0.647	0.332
Co-Tuning	0.898	0.038	[0.793, 0.957]	0.699	medium	0.408	0.021
DELTA	0.893	0.040	[0.676, 0.948]	0.788	medium	0.948	0.003
SpotTune	0.889	0.020	[0.773, 0.959]	1.365	large	0.963	0.001
BSS	0.881	0.044	[0.753, 0.953]	0.947	large	0.899	0.093
Bi-Tuning	0.878	0.033	[0.775, 0.948]	1.276	large	0.934	0.001
MultiTune	0.849	0.051	[0.766, 0.939]	1.390	large	1.000	0.000
MARS-PGM	0.802	0.037	[0.473, 0.902]	2.898	large	1.000	0.000

MAD: mean absolute deviation of the median; CI: confidence interval; *γ*: Akinshin’s gamma.

**Table 6:** Decision matrix for the pair-wise comparison of the algorithms. The algorithms are sorted by descending median performance.

Algorithm	L^2^-SP	Fine-Tuning	Co-Tuning	DELTA	Spot-Tune	BSS	Bi-Tuning	MultiTune	MARS-PGM
L^2^-SP	—	^ns^	^ns^	≪ *	≪ **	^ns^	≪ *	≪ ***	≪ ***
Fine-Tuning	^ns^	—	^ns^	^ns^	≪ *	^ns^	≪ *	≪ ***	≪ ***
Co-Tuning	^ns^	^ns^	—	≪ *	≪ **	≪ *	^ns^	≪ ***	≪ ***
DELTA	≫ *	^ns^	≫ *	—	^ns^	^ns^	^ns^	^ns^	≪ ***
SpotTune	≫ **	≫ *	≫ **	^ns^	—	^ns^	^ns^	≪ ***	≪ ***
BSS	^ns^	^ns^	≫ *	^ns^	^ns^	—	^ns^	≪ ***	≪ ***
Bi-Tuning	≫ *	≫ *	^ns^	^ns^	^ns^	^ns^	—	≪ ***	≪ ***
MultiTune	≫ ***	≫ ***	≫ ***	^ns^	≫ ***	≫ ***	≫ ***	—	≪ ***
MARS-PGM	≫ ***	≫ ***	≫ ***	≫ ***	≫ ***	≫ ***	≫ ***	≫ ***	—

≪: negative mean difference; ≫: positive mean difference; *: *P*(·) ≥ 0.9; **: *P*(·) ≥ 0.95; ***: *P*(·) ≥ 0.99; ^ns^: not significant.

**Table 7:** Decision matrix for the pair-wise comparison of vanilla fine-tuning with all other algorithms on the task level.

Task	BSS	Bi-Tuning	Co-Tuning	DELTA	L^2^-SP	MARS-PGM	MultiTune	Spot-Tune
#1	≪ **, l	≪ *, l	^ns^	^ns^	^ns^	≪ ***, l	≪ ***, l	≪ ***, l
#2	^ns^	^ns^	^ns^	≪ **, l	^ns^	≪ **, l	≪ *, l	^ns^
#3	^ns^	≪ ***, l	^ns^	^ns^	≫ *, l	≪ *, l	≪ *, l	≪ **, l
#4	≪ **, l	^ns^	^ns^	≫ *, l	^ns^	^ns^	≪ *, l	^ns^
#5	≪ **, m	≪ *, l	≪ **, l	^ns^	^ns^	≪ **, l	≪ **, l	^ns^
#6	^ns^	^ns^	^ns^	^ns^	^ns^	^ns^	^ns^	^ns^
#7	^ns^	^ns^	^ns^	^ns^	^ns^	≪ *, l	≪ **, l	≪ ***, l
#8	^ns^	^ns^	^ns^	^ns^	^ns^	^ns^	^ns^	^ns^
#9	^ns^	≫ ***, l	≫ *, l	≫ *, l	^ns^	≪ *, l	≪ *, l	^ns^
#10	^ns^	^ns^	^ns^	^ns^	^ns^	^ns^	^ns^	^ns^
#11	^ns^	^ns^	≫ *, l	≫ *, l	^ns^	^ns^	^ns^	≫ *, m
#12	^ns^	^ns^	≫ *, l	^ns^	^ns^	^ns^	^ns^	≫ **, l

≪: negative mean difference; ≫: positive mean difference; *: *P*(·) ≥ 0.9; **: *P*(·) ≥ 0.95; ***: *P*(·) ≥ 0.99; ^ns^: not significant; m: medium effect; l: large effect. Significant positive mean differences (*P*(≫) ≥ 0.9) are additionally highlighted in bold.

**Table 8:** Mean and standard deviation of the AUC for fine-tuning, L^2^-SP, and Co-Tuning relative to different dataset size settings.

Dataset	Baseline	Distance Regularization	Shared Domains
	Fine-Tuning	L^2^-SP	Co-Tuning
Base	0.957 ± 0.016	0.955 ± 0.022	0.958 ± 0.014
Large	0.961 ± 0.009	0.960 ± 0.011	0.966 ± 0.010
XLarge	0.962 ± 0.013	0.966 ± 0.009	0.971 ± 0.007
Median	0.961	0.960	0.966

**Table 9:** Mean and standard deviation of the balanced accuracy for fine-tuning, L^2^-SP, and Co-Tuning relative to different dataset size settings.

Dataset	Baseline	Distance Regularization	Shared Domains
	Fine-Tuning	L^2^-SP	Co-Tuning
Base	0.782 ± 0.050	0.796 ± 0.065	0.787 ± 0.048
Large	0.802 ± 0.032	0.797 ± 0.035	0.818 ± 0.032
XLarge	0.807 ± 0.036	0.819 ± 0.028	0.832 ± 0.023
Median	0.802	0.797	0.818

**Table 10:** Mean and standard deviation of the F1 score for fine-tuning, L^2^-SP, and Co-Tuning relative to different dataset size settings.

Dataset	Baseline	Distance Regularization	Shared Domains
	Fine-Tuning	L^2^-SP	Co-Tuning
Base	0.776 ± 0.055	0.791 ± 0.072	0.781 ± 0.055
Large	0.798 ± 0.034	0.794 ± 0.037	0.815 ± 0.036
XLarge	0.803 ± 0.037	0.817 ± 0.029	0.829 ± 0.023
Median	0.798	0.794	0.815

**Table 11:** Descriptive statistics for the ranked algorithms using the dataset size “Base”.

Algorithm	Mean	STD	CI	*d*	Magnitude	*P*(≪)	*P*(=)
Co-Tuning	0.958	0.014	[0.949, 0.967]	–	–	–	–
Fine-Tuning	0.957	0.016	[0.947, 0.967]	0.079	negligible	0.473	0.241
L^2^-SP	0.955	0.022	[0.941, 0.969]	0.197	negligible	0.596	0.173

STD: standard deviation; CI: confidence interval; *d*: Cohen’s *d*.

**Table 12:** Decision matrix for the pair-wise Bayesian correlated *t*-test of algorithms using the dataset size “Base”.

Algorithm	Fine-Tuning	L^2^-SP	Co-Tuning
Fine-Tuning	—	^ns^	^ns^
L^2^-SP	^ns^	—	^ns^
Co-Tuning	^ns^	^ns^	—

^ns^: not significant.

**Table 13:** Descriptive statistics for the ranked algorithms using the dataset size “Large”.

Algorithm	Mean	STD	CI	*d*	Magnitude	*P*(≪)	*P*(=)
Co-Tuning	0.966	0.010	[0.959, 0.972]	–	–	–	–
Fine-Tuning	0.961	0.009	[0.955, 0.966]	0.518	medium	0.830	0.088
L^2^-SP	0.960	0.011	[0.953, 0.967]	0.544	medium	0.849	0.081

STD: standard deviation; CI: confidence interval; *d*: Cohen’s *d*.

**Table 14:** Decision matrix for the pair-wise Bayesian correlated *t*-test of algorithms using the dataset size “Large”.

Algorithm	Fine-Tuning	L^2^-SP	Co-Tuning
Fine-Tuning	—	^ns^	^ns^
L^2^-SP	^ns^	—	^ns^
Co-Tuning	^ns^	^ns^	—

^ns^: not significant.

**Table 15:** Descriptive statistics for the ranked algorithms using the dataset size “XLarge”.

Algorithm	Median	MAD	CI	*γ*	Magnitude	*P*(≪)	*P*(=)
Co-Tuning	0.972	0.006	[0.964, 0.981]	–	–	–	–
L^2^-SP	0.967	0.005	[0.958, 0.976]	0.619	medium	0.876	0.063
Fine-Tuning	0.965	0.006	[0.951, 0.979]	0.748	medium	0.951	0.025

MAD: mean absolute deviation of the median; CI: confidence interval; *γ*: Akinshin’s gamma.

**Table 16:** Decision matrix for the pair-wise Bayesian correlated *t*-test of algorithms using the dataset size “XLarge”.

Algorithm	Fine-Tuning	L^2^-SP	Co-Tuning
Fine-Tuning	—	^ns^	≫ **, m
L^2^-SP	^ns^	—	^ns^
Co-Tuning	≪ **, m	^ns^	—

≪: negative mean difference; ≫: positive mean difference; **: *P*(·) ≥ 0.95; ^ns^: not significant; m: medium effect.

## Data Availability

Publicly available datasets were analyzed in this study. The BreCaHAD dataset is available from Figshare at https://doi.org/10.6084/m9.figshare.7379186 (accessed on 9 March 2023). The TUPAC16 dataset can be found at: https://tupac.grand-challenge.org/ (accessed on 9 March 2023). The CAMELYON17 dataset can be found at: https://camelyon17.grand-challenge.org/ (accessed on 7 March 2023). The dataset for TIL classification is available from Zenodo at https://zenodo.org/record/6604094 (accessed on 28 June 2023). The CRC-5000 dataset is available from Zenodo at https://zenodo.org/record/53169 (accessed on 9 March 2023). The DRCO dataset (K. Lindman, M. Lindvall, C. B. Stadler, C. Lundstrom, and D. Treanor, 2019, Colon data from the Visual Sweden project DROID) is available upon request at https://datahub.aida.scilifelab.se/10.23698/aida/drco (accessed on 4 April 2023). The Queensland dataset is available from UQ eSpace at https://espace.library.uq.edu.au/view/UQ:8be4bd0 (accessed on 8 March 2023). The DRSK dataset (K. Lindman, J. F. Rose, M. Lindvall, and C. B. Stadler, 2019, Skin data from the Visual Sweden project DROID) is available upon request at https://datahub.aida.scilifelab.se/10.23698/aida/drsk (accessed on 4 April 2023).

## Appendix B

**Table A1:** Posterior matrix with probabilities from the pair-wise Bayesian signed-rank test for comparisons across all histopathological tasks.

**Algorithm**	**L^2^-SP**			**Fine-Tuning**			**Co-Tuning**		
	***P*** **(** **≪** **)**	***P*(=)**	***P*** **(** **≫** **)**	***P*** **(** **≪** **)**	***P*(=)**	***P*** **(** **≫** **)**	***P*** **(** **≪** **)**	***P*(=)**	***P*** **(** **≫** **)**
L^2^-SP	—	—	—	0.647	0.332	0.021	0.408	0.021	0.571
Fine-Tuning	0.021	0.332	0.647	0.647	0.332	0.021	0.093	0.553	0.353
Co-Tuning	0.571	0.021	0.408	—	—	—	—	—	—
DELTA	0.049	0.003	**0.948**	0.353	0.553	0.093	0.078	0.011	**0.911**
SpotTune	0.036	0.001	**0.963**	0.130	0.021	0.849	0.013	0.001	**0.987**
BSS	0.009	0.093	0.899	0.086	0.001	**0.912**	0.000	0.069	**0.931**
Bi-Tuning	0.064	0.001	**0.934**	0.004	0.588	0.408	0.001	0.115	0.884
MultiTune	0.000	0.000	**1.000**	0.062	0.021	**0.917**	0.000	0.000	**1.000**
MARS-PGM	0.000	0.000	**1.000**	0.001	0.000	**0.999**	0.000	0.000	**1.000**
**Algorithm**	**DELTA**			**SpotTune**			**BSS**		
	***P*** **(** **≪** **)**	***P*(=)**	***P*** **(** **≫** **)**	***P*** **(** **≪** **)**	***P*(=)**	***P*** **(** **≫** **)**	***P*** **(** **≪** **)**	***P*(=)**	***P*** **(** **≫** **)**
L^2^-SP	**0.948**	0.003	0.049	**0.963**	0.001	0.036	0.899	0.093	0.009
Fine-Tuning	0.849	0.021	0.130	**0.912**	0.001	0.086	0.408	0.588	0.004
Co-Tuning	**0.911**	0.011	0.078	**0.987**	0.001	0.013	**0.931**	0.069	0.000
DELTA	—	—	—	0.490	0.000	0.509	0.288	0.027	0.685
SpotTune	0.509	0.000	0.490	—	—	—	0.306	0.001	0.693
BSS	0.685	0.027	0.288	0.693	0.001	0.306	—	—	—
Bi-Tuning	0.544	0.007	0.449	0.460	0.001	0.539	0.237	0.281	0.482
MultiTune	0.103	0.000	0.897	0.000	0.000	**0.999**	0.000	0.001	**0.999**
MARS-PGM	0.002	0.000	**0.998**	0.000	0.000	**1.000**	0.000	0.000	**1.000**
**Algorithm**	**Bi-Tuning**			**MultiTune**			**MARS-PGM**		
	***P*** **(** **≪** **)**	***P*(=)**	***P*** **(** **≫** **)**	***P*** **(** **≪** **)**	***P*(=)**	***P*** **(** **≫** **)**	***P*** **(** **≪** **)**	***P*(=)**	***P*** **(** **≫** **)**
L^2^-SP	**0.934**	0.001	0.064	**1.000**	0.000	0.000	**1.000**	0.000	0.000
Fine-Tuning	**0.917**	0.021	0.062	**0.999**	0.000	0.001	**1.000**	0.000	0.000
Co-Tuning	0.884	0.115	0.001	**1.000**	0.000	0.000	**1.000**	0.000	0.000
DELTA	0.449	0.007	0.544	0.897	0.000	0.103	**0.998**	0.000	0.002
SpotTune	0.539	0.001	0.460	**0.999**	0.000	0.000	**1.000**	0.000	0.000
BSS	0.482	0.281	0.237	**0.999**	0.001	0.000	**1.000**	0.000	0.000
Bi-Tuning	—	—	—	**0.995**	0.005	0.000	**1.000**	0.000	0.000
MultiTune	0.000	0.005	**0.995**	—	—	—	**0.998**	0.000	0.002
MARS-PGM	0.000	0.000	**1.000**	0.002	0.000	**0.998**	—	—	—

*P*(≪): probability of negative mean difference; *P*(=): probability of equivalent results; *P*(≫): probability of positive mean difference. Significant values (*P*(·) ≥ 0.9) are highlighted in bold.

## Appendix C

**Table A2:** Posterior matrix with probabilities from the pair-wise Bayesian correlated *t*-test and effect size for comparison with fine-tuning, separated by task.

**Task**	**BSS**				**Bi-Tuning**				**Co-Tuning**			
	***P*** **(** **≪** **)**	***P*(=)**	***P*** **(** **≫** **)**	***d*** **/*γ***	***P*** **(** **≪** **)**	***P*(=)**	***P*** **(** **≫** **)**	***d*** **/*γ***	***P*** **(** **≪** **)**	***P*(=)**	***P*** **(** **≫** **)**	***d*** **/*γ***
1	0.966	0.013	0.021	−1.171	0.910	0.018	0.073	−1.845	0.466	0.072	0.463	−0.004
2	0.705	0.059	0.236	−0.928	0.865	0.042	0.093	−0.755	0.293	0.044	0.664	0.653
3	0.707	0.110	0.183	−0.378	0.996	0.001	0.003	−1.801	0.172	0.181	0.648	0.222
4	0.979	0.005	0.016	−2.452	0.799	0.045	0.156	−0.955	0.871	0.035	0.094	−1.080
5	0.964	0.019	0.017	−0.780	0.931	0.015	0.054	−1.861	0.976	0.009	0.015	−1.241
6	0.226	0.340	0.434	0.065	0.274	0.258	0.468	0.077	0.490	0.170	0.340	−0.089
7	0.313	0.170	0.517	0.119	0.615	0.092	0.294	−0.324	0.493	0.157	0.351	−0.091
8	0.468	0.085	0.446	0.048	0.360	0.075	0.564	−0.379	0.544	0.067	0.388	0.102
9	0.385	0.126	0.489	0.083	0.004	0.003	**0.993**	1.407	0.037	0.016	**0.947**	1.379
10	0.643	0.080	0.277	−0.411	0.739	0.070	0.191	−0.598	0.591	0.110	0.299	−0.250
11	0.408	0.098	0.494	0.087	0.133	0.046	0.822	0.919	0.067	0.023	**0.910**	1.406
12	0.231	0.076	0.693	0.510	0.337	0.082	0.581	0.284	0.048	0.009	**0.942**	2.623
**Task**	**DELTA**				**L^2^-SP**				**MARS-PGM**			
	***P*** **(** **≪** **)**	***P*(=)**	***P*** **(** **≫** **)**	***d*** **/*γ***	***P*** **(** **≪** **)**	***P*(=)**	***P*** **(** **≫** **)**	***d*** **/*γ***	***P*** **(** **≪** **)**	***P*(=)**	***P*** **(** **≫** **)**	***d*** **/*γ***
1	0.891	0.023	0.087	−1.579	0.515	0.140	0.345	-0.121	0.997	0.000	0.003	−6.447
2	0.984	0.001	0.016	−16.406	0.411	0.043	0.546	0.180	0.951	0.010	0.039	−1.374
3	0.510	0.116	0.374	−0.116	0.051	0.033	**0.917**	0.879	0.920	0.019	0.061	−1.540
4	0.043	0.020	**0.937**	1.230	0.089	0.054	0.857	0.707	0.894	0.020	0.085	−1.752
5	0.493	0.209	0.298	−0.094	0.444	0.109	0.447	0.002	0.953	0.010	0.037	−2.102
6	0.599	0.157	0.244	−0.209	0.058	0.052	0.890	0.626	0.840	0.034	0.127	−1.234
7	0.841	0.038	0.121	−1.086	0.668	0.120	0.212	−0.323	0.941	0.013	0.046	−1.908
8	0.081	0.023	0.896	1.692	0.653	0.067	0.279	−0.769	0.821	0.026	0.153	−0.821
9	0.038	0.025	**0.937**	0.938	0.238	0.138	0.624	0.251	0.943	0.012	0.044	−1.967
10	0.828	0.044	0.128	−0.957	0.783	0.057	0.160	−0.760	0.871	0.029	0.100	−1.332
11	0.038	0.024	**0.937**	0.986	0.092	0.158	0.750	0.272	0.549	0.086	0.365	−0.209
12	0.669	0.085	0.246	−0.433	0.258	0.100	0.642	0.343	0.177	0.049	0.774	0.878
**Task**	**MultiTune**				**SpotTune**							
	***P*** **(** **≪** **)**	***P*(=)**	***P*** **(** **≫** **)**	***d*** **/*γ***	***P*** **(** **≪** **)**	***P*(=)**	***P*** **(** **≫** **)**	***d*** **/*γ***				
1	0.994	0.001	0.005	−4.330	0.991	0.002	0.008	−3.048				
2	0.931	0.010	0.059	−1.493	0.849	0.023	0.128	−2.275				
3	0.909	0.024	0.067	−1.347	0.962	0.012	0.027	−1.480				
4	0.914	0.016	0.069	−1.949	0.739	0.074	0.187	−0.571				
5	0.988	0.002	0.010	−2.851	0.820	0.037	0.143	−1.144				
6	0.528	0.065	0.406	−0.185	0.106	0.060	0.834	0.682				
7	0.961	0.010	0.029	−1.887	0.999	0.000	0.001	−2.932				
8	0.705	0.015	0.281	−0.384	0.789	0.048	0.162	−1.139				
9	0.924	0.026	0.050	−1.086	0.461	0.074	0.465	0.006				
10	0.811	0.037	0.152	−1.144	0.471	0.128	0.401	−0.056				
11	0.264	0.062	0.673	0.573	0.045	0.042	**0.912**	0.670				
12	0.163	0.062	0.775	0.690	0.012	0.005	**0.983**	1.804				

*P*(≪): probability of negative mean difference; *P*(=): probability of equivalent results; *P*(≫): probability of positive mean difference; *d*: Cohen’s *d*; *γ*: Akinshin’s gamma. Significant positive mean differences (*P*(·) ≥ 0.9) are highlighted in bold.

## Appendix E

**Table A3:** Posterior matrix for the dataset size “Base” with probabilities from the pair-wise Bayesian correlated *t*-test and effect size.

Algorithm	Fine-Tuning				L^2^-SP				Co-Tuning			
	*P*(≪)	*P*(=)	*P*(≫)	*d*	*P*(≪)	*P*(=)	*P*(≫)	*d*	*P*(≪)	*P*(=)	*P*(≫)	*d*
Fine-Tuning	—	—	—	—	0.525	0.173	0.302	−0.127	0.286	0.241	0.473	0.079
L^2^-SP	0.302	0.173	0.525	0.127	—	—	—	—	0.231	0.173	0.596	0.197
Co-Tuning	0.473	0.241	0.286	−0.079	0.596	0.173	0.231	−0.197	—	—	—	—

*P*(≪): probability of negative mean difference; *P*(=): probability of equivalent results; *P*(≫): probability of positive mean difference; *d*: Cohen’s *d*. Significant values (*P*(·) ≥ 0.9) are highlighted in bold.

**Table A4:** Posterior matrix for the dataset size “Large” with probabilities from the pair-wise Bayesian correlated *t*-test and effect size.

Algorithm	Fine-Tuning				L^2^-SP				Co-Tuning			
	*P*(≪)	*P*(=)	*P*(≫)	*d*	*P*(≪)	*P*(=)	*P*(≫)	*d*	*P*(≪)	*P*(=)	*P*(≫)	*d*
Fine-Tuning	—	—	—	—	0.476	0.170	0.355	−0.072	0.082	0.088	0.830	0.518
L^2^-SP	0.355	0.170	0.476	0.072	—	—	—	—	0.070	0.081	0.849	0.544
Co-Tuning	0.830	0.088	0.082	−0.518	0.849	0.081	0.070	−0.544	—	—	—	—

*P*(≪): probability of negative mean difference; *P*(=): probability of equivalent results; *P*(≫): probability of positive mean difference; *d*: Cohen’s *d*. Significant values (*P*(·) ≥ 0.9) are highlighted in bold.

**Table A5:** Posterior matrix for the dataset size “XLarge” with probabilities from the pair-wise Bayesian correlated *t*-test and effect size.

Algorithm	Fine-Tuning				L^2^-SP				Co-Tuning			
	*P*(≪)	*P*(=)	*P*(≫)	*γ*	*P*(≪)	*P*(=)	*P*(≫)	*γ*	*P*(≪)	*P*(=)	*P*(≫)	*γ*
Fine-Tuning	—	—	—	—	0.198	0.099	0.703	0.164	0.024	0.025	**0.951**	0.748
L^2^-SP	0.703	0.099	0.198	−0.164	—	—	—	—	0.061	0.063	0.876	0.619
Co-Tuning	**0.951**	0.025	0.024	−0.748	0.876	0.063	0.061	−0.619	—	—	—	—

*P*(≪): probability of negative mean difference; *P*(=): probability of equivalent results; *P*(≫): probability of positive mean difference; *γ*: Akinshin’s gamma. Significant values (*P*(·) ≥ 0.9) are highlighted in bold.
